# Phospholipid-grafted PLLA electrospun micro/nanofibers immobilized with small extracellular vesicles from rat adipose mesenchymal stem cells promote wound healing in diabetic rats

**DOI:** 10.1093/rb/rbac071

**Published:** 2022-09-26

**Authors:** Jing Li, Shunshun Yan, Weiju Han, Zixuan Dong, Junliang Li, Qi Wu, Xiaoling Fu

**Affiliations:** School of Materials Science and Engineering, South China University of Technology, Guangzhou 510006, China; National Engineering Research Center for Tissue Restoration and Reconstruction and Innovation Center for Tissue Restoration and Reconstruction, Guangzhou 510006, China; Laboratory of Biomedical Engineering of Guangdong Province, South China University of Technology, Guangzhou 510006, China; National Engineering Research Center for Tissue Restoration and Reconstruction and Innovation Center for Tissue Restoration and Reconstruction, Guangzhou 510006, China; Laboratory of Biomedical Engineering of Guangdong Province, South China University of Technology, Guangzhou 510006, China; School of Biomedical Sciences and Engineering, South China University of Technology, Guangzhou International Campus, Guangzhou 511442, China; National Engineering Research Center for Tissue Restoration and Reconstruction and Innovation Center for Tissue Restoration and Reconstruction, Guangzhou 510006, China; Laboratory of Biomedical Engineering of Guangdong Province, South China University of Technology, Guangzhou 510006, China; School of Biomedical Sciences and Engineering, South China University of Technology, Guangzhou International Campus, Guangzhou 511442, China; National Engineering Research Center for Tissue Restoration and Reconstruction and Innovation Center for Tissue Restoration and Reconstruction, Guangzhou 510006, China; Laboratory of Biomedical Engineering of Guangdong Province, South China University of Technology, Guangzhou 510006, China; School of Biomedical Sciences and Engineering, South China University of Technology, Guangzhou International Campus, Guangzhou 511442, China; National Engineering Research Center for Tissue Restoration and Reconstruction and Innovation Center for Tissue Restoration and Reconstruction, Guangzhou 510006, China; Laboratory of Biomedical Engineering of Guangdong Province, South China University of Technology, Guangzhou 510006, China; School of Biomedical Sciences and Engineering, South China University of Technology, Guangzhou International Campus, Guangzhou 511442, China; National Engineering Research Center for Tissue Restoration and Reconstruction and Innovation Center for Tissue Restoration and Reconstruction, Guangzhou 510006, China; Laboratory of Biomedical Engineering of Guangdong Province, South China University of Technology, Guangzhou 510006, China; School of Biomedical Sciences and Engineering, South China University of Technology, Guangzhou International Campus, Guangzhou 511442, China; School of Biomedical Sciences and Engineering, South China University of Technology, Guangzhou International Campus, Guangzhou 511442, China

**Keywords:** chronic wound healing, small extracellular vesicles, sustained release, DSPE-PLLA fibers

## Abstract

Small extracellular vesicles (sEVs) derived from mesenchymal stem cells (MSCs) can deliver a variety of bioactive factors to create a favorable local microenvironment, thereby holding huge potential in chronic wound repair. However, free sEVs administrated intravenously or locally are usually cleared rapidly, resulting in an insufficient duration of the efficacy. Thus, strategies that enable optimized retention and release profiles of sEVs at wound sites are desirable. Herein, we fabricated novel functional phosphoethanolamine phospholipid-grafted poly-l-lactic acid micro/nanofibers (DSPE-PLLA) to carry and retain sEVs from rat adipose MSCs, enabling the slow local release of sEVs. Our results showed that sEVs@DSPE-PLLA promoted the proliferation, migration and gene expression (Col I, Col III, TGF-β, α-SMA, HIF-1α) of fibroblasts. It also promoted keratinocyte proliferation. In addition, sEVs@DSPE-PLLA helped polarize macrophages toward the M2 phenotype by increasing the expression of anti-inflammatory genes (Arginase 1, CD 206, IL-10) and inhibiting the expression of pro-inflammatory genes (IL-1β, TNF-α). Further in vivo study in diabetic rat models showed that sEVs@DSPE-PLLA improved the wound-healing process by alleviating the inflammatory responses, stimulating cell proliferation, collagen deposition and angiogenesis. These results highlight the potential of using DSPE-grafted scaffolds for extracellular vesicle immobilization and suggest sEVs@DSPE-PLLA micro/nanofibers as promising functional wound dressings for diabetic wounds.

## Graphical Abstract

**Figure:**
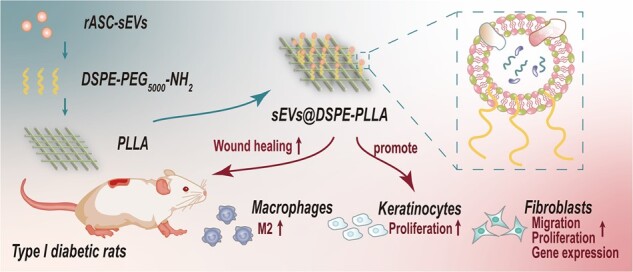

## Introduction

Wound healing is a dynamic and interactive process, which requires various cells, chemokines and cytokines [1, 2] to help the process of inflammation, new tissue formation and eventual wound closure [3]. With the high prevalence of diabetes mellitus all around the world, complications such as chronic wounds have been a major threat to patients and bring a heavy economic burden [4, 5]. In diabetic chronic wounds, severe imbalance in the local physiological environment, which leads to multiple pathological changes of the wound tissue and disturbs the normal repair process, is one of the key reasons for the poor healing outcome. For instance, higher levels of multiple inflammatory cytokines, including tumor necrosis factor α (TNF-α), granulocyte colony-stimulating factor and monocyte chemoattractant protein-1, are detected at diabetic wound sites [6–8]. Meanwhile, the levels of many growth factors and cytokines, including epidermal growth factor, fibroblast growth factor, macrophage inflammatory protein-1 α (MIP-1α) and MIP-1b, are reduced. As a result, a variety of cell functions are impaired. For example, abnormal endothelial cell metabolism leads to vascular insufficiency [9]. The migration of keratinocytes is blocked and the epidermal structure cannot be restored promptly [10–12]. Fibroblasts at the wound edge become senescence, exhibiting limited migratory ability and weakened response to growth factor signals [13]. Besides, due to the elevated inflammatory cytokines, inflammatory cells continue to flood into the wound and cause undesirable damage to the tissue. Therefore, delivery of a single bioactive factor to the diabetic wound sites can hardly ameliorate the microenvironment and improve the healing outcomes [6].

Mesenchymal stem cells (MSCs) have been shown to promote skin tissue repair [14, 15], but their application has been limited by the relatively low survival rate and chromosomal variation [16]. Recently, accumulated evidence demonstrates that MSCs exert their therapeutical effects mainly through the paracrine mechanism, that is, secreting various bioactive factors to create a favorable local microenvironment [17, 18]. One important kind of MSCs paracrine product is the small extracellular vesicles (sEVs), which are small membrane particles with a diameter of <200 nm [19–21]. sEVs contain a complex cargo rich in proteins, lipids, RNAs (e.g. microRNAs, lncRNAs and mRNAs) and DNAs and thus can modify the microenvironment and regulate cells in the neighborhood. Compared to MSC treatment, MSC-sEVs have several potential advantages, such as lower immunogenicity and higher stability, and no potential to transdifferentiate into a different cell type [22]. Many studies have confirmed that MSC-sEVs benefit many physiological processes in wound healing, such as helping immunoregulation [23, 24], inducing angiogenesis [25] and accelerating re-epithelialization [26], making them attractive candidates for skin tissue repair. However, in most cases, free sEVs were given intravenously, which leads to rapid clearance *in vivo*. Besides, the systemic administration of sEVs leads to an accumulation of sEVs in the spleen and liver [8, 27, 28]. As a result, very few sEVs reach the wound sites, which severely limits their clinical use. Local delivery can increase the concentration of sEVs at wound sites. Nevertheless, the sEVs are usually cleared very quickly due to the high metabolic activity of wounds. Thus, strategies that enable optimized retention and release profiles of sEVs at wound sites are desirable.

**Figure 1.**
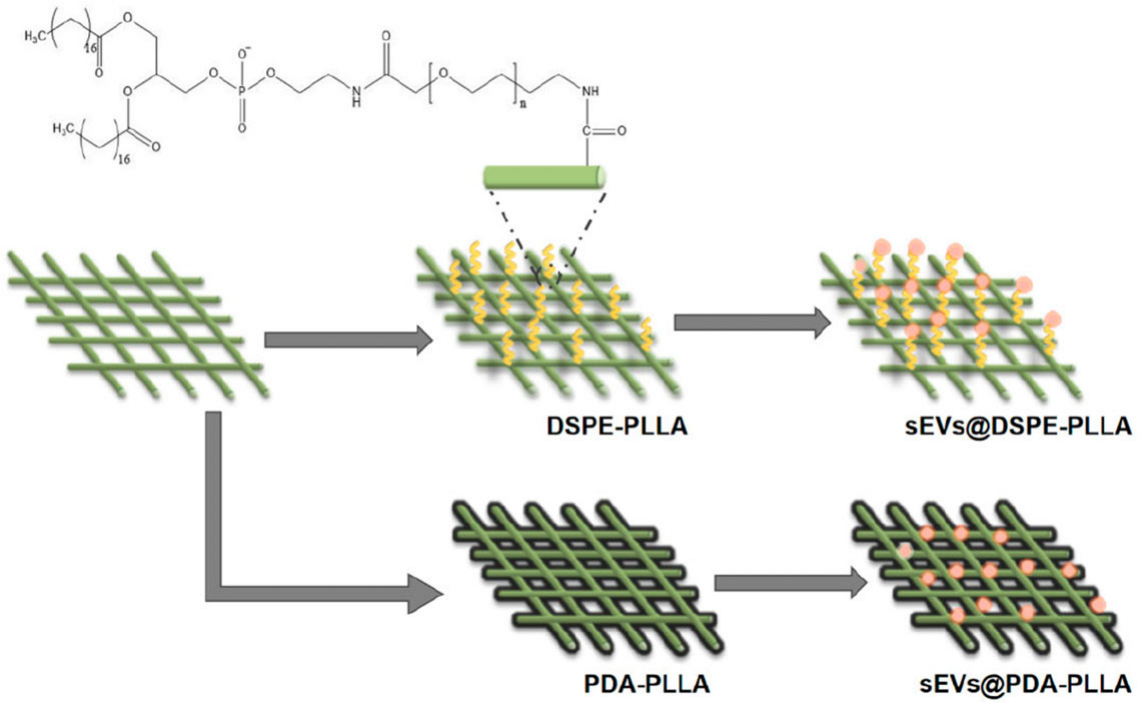
Schematic illustration of the fabrication of sEVs@PDA-PLLA and sEVs@DSPE-PLLA.

Herein, we propose to immobilize sEVs onto electrospun micro/nanofibers, which have been widely used as wound dressings because of their similarity with skin extracellular matrix (ECM) in morphological and dimensional features [29, 30]. sEVs isolated from adipose-derived MSCs (ASCs) were used in this study because ASCs are easy to obtain and have a rich source in the body [31]. Two strategies were selected for the immobilization of sEVs on electrospun micro/nanofibers. The first one is to deposit a polydopamine (PDA) layer on the surface of electrospun fibers (sEVs@PDA-PLLA). This mussel-inspired immobilization strategy has been proved to be effective in loading bioactive factors on almost all materials and exhibits good biocompatibility [32]. The second one is to graft PEG-phospholipids (DSPE-PEG_5000_-NH_2_) on the surface of electrospun fibers (sEVs@DSPE-PLLA). Many studies have shown that lipid chains could insert into the cell membrane through the hydrophobic interaction with the phospholipids of the plasma membrane [33–35]. Since sEVs had a lipid bilayer similar to cells, we hypothesize that the conjugated phospholipids on the surfaces of the fibers are able to insert into the membrane of sEVs, thereby immobilizing the sEVs (Fig. 1). The biological functions of functional electrospun fibrous wound dressings were evaluated at the molecular, cellular and animal levels to investigate their regulation on diabetic chronic wounds in rats.

## Materials and methods

### Cell culture

Rat ASCs (p3–p7) were isolated from 6-week-old rats and cultivated in α-MEM medium (Gibco, USA) with 10% fetal bovine serum (FBS, Gibco) and 1% penicillin–streptomycin (Gibco). When cell confluence reached 80%, the medium was replaced with sEV-free medium which contained 90% α-MEM and 10% sEV-free serum (System Biosciences, USA) and 1% penicillin–streptomycin (Gibco). Human foreskin fibroblasts (p10–p15) were propagated in DMEM (4.5 g/L l-glucose, Gibco) with 10% FBS and 1% penicillin–streptomycin. Human Neonatal Foreskin Epidermal Keratinocytes (p4–p7, ATCC, USA) were grown in keratinocytes culture medium, consisting of Dermal Cell Basal Medium supplemented with Keratinocyte Growth Kit and Penicillin–Streptomycin–Amphotericin B Solution (ATCC). Macrophages were cultured in DMEM with 10% FBS and 1% penicillin–streptomycin. All the cells were cultured in a humidified 37°C, 5% CO_2_ incubator (Thermo Scientific, USA) and refreshed medium every 3 days until the confluence of the cells reached 80% in this study.

### Isolation and characterization of rat adipose-derived mesenchymal stem cell-sEVs

After culturing rat adipose-derived mesenchymal stem cells (rASCs) with sEV-free medium for 48 h, the culture supernatant was collected. The sEVs derived from rASCs (rASC-sEVs) were isolated by differential ultracentrifugation as described previously [36]. In brief, the collected supernatant was centrifuged sequentially at 300 × *g* for 10 min, 2,000 × *g* for 10 min and 10 000 × *g* for 30 min to remove cells, dead cells, apoptotic bodies and cell debris. Then, the supernatant was centrifuged at 100 000 × *g* for 70 min to harvest rASC-sEVs. The pellets were further purified by re-suspension in phosphate-buffered saline (PBS, Gibco) and centrifugation at 100 000 × g for 70 min to remove contaminating protein. The obtained rASC-sEVs were suspended in PBS and stored at −80°C for later use. The total protein content of rASC-sEVs was quantified by BCA Protein Assay Kit (Beyotime, Biotechnology) according to the manufacturer’s instructions. The rASC-sEVs were sterilized by the Centrifugal Filter Units 0.22-μm GV Durapore 50^®^ (Merck, USA). The morphology of rASC-sEVs was examined by transmission electron microscopy (TEM, ZetaView Particle, PMX, Germany), and the particle size of rASC-sEVs was detected by nanoparticle tracking analysis (NTA, Tecnai G2 Spirit, FEI, USA). Western blotting was used to detect the specific surface markers of rASC-sEVs, including Tsg101 (1:5000, Abcam), CD9 (1:2000, Abcam), CD63 (1:2000, Abcam) and Calnexin (1:2000, Abcam).

### Fabrication of electrospun fibrous wound dressings

PLLA micro/nanofibrous wound dressings (PLLA) were fabricated by electrospinning. In short, PLLA (Mn = 100 000, China) was dissolved in hexafluoroisopropanol (Aladdin, China) at 10% (w/v) concentration and stirred for at least 6 h. Under the voltage of 12 kV, the polymer solution was extruded with a syringe pump through a 21-G needle. The micro/nanofibers were received with tinfoil and had a thickness of approximately 300 μm. Then, the fibers were dried for 48 h under vacuum to remove the residual organic solvent. Polydopamine modified PLLA fibrous wound dressings (PDA-PLLA) were obtained by soaking in Tris-HCl solution (pH = 8.5, 1 mol/l) with dopamine hydrochloride (2 mg/ml) at room temperature for 4 h. To get PEG-phospholipid-grafted PLLA fibrous wound dressings (DSPE-PLLA), PLLA were soaked in NaOH (0.1 M) for 20 min to expose the carboxyl and then immersed in 4-Morpholineethanesulfonic acid (MES) solution (pH = 5.6, Thermo Fisher Scientific, USA) containing EDC (0.05 mg/ml, Aladdin) and NHS (0.025 mg/ml, Aladdin) for 30 min to activate the exposed carboxyl. At last, PLLA were immersed overnight in MES solution containing 1,2-distearoyl-*sn*-glycero-3-phosphoethanolamine-*N*-[amino(polyethyleneglycol)] (DSPE-PEG_5000_-NH2) (10 mg/ml, Beijing Hua Wei Rui Ke Chemical) to graft DSPE-PEG5000-NH2 on the surface of PLLA fibers. All of the above procedures were conducted at room temperature. All the wound dressings were sterilized by immersing in 75% ethanol for 1 h and exposed to UV light for 2 h before use. The rASC-sEVs were immobilized onto the fibers by incubating the fibers with sterilized rASC-sEVs at 4°C for 6 h. Then, excess rASC-sEVs were washed off with PBS. PLLA, PDA-PLLA and DSPE-PLLA wound dressings loaded with rASC-sEVs were thereafter referred to as sEVs@PLLA, sEVs@PDA-PLLA and sEVs@DSPE-PLLA, respectively.

### Characterization of electrospun fibrous wound dressings

The structure and morphology of PLLA, PDA-PLLA and DSPE-PLLA were observed by emission scanning electron microscope (SEM). The average fiber diameter was determined using ImageJ according to the representative SEM images. The formation of amide(–CONH) in DSPE-PLLA was proved by Fourier transform infrared spectroscopy (FTIR; Vector 33, Bruker, Germany).

For rASC-sEVs loading assay, the total amount of rASC-sEVs in PBS was determined by BCA Protein Assay Kit and then loaded onto PLLA, PDA-PLLA and DSPE-PLLA. Then, the amount of free rASC-sEVs in PBS was determined. The loading capacity was calculated by total sEVs minus free rASC-sEVs in PBS. To determine the release profile of rASC-sEVs, the rASC-sEVs were first labeled with PKH26 (Sigma) according to the manufacturer’s protocol. In short, PKH26 was co-incubated with rASC-sEVs for 4 min and then stopped by 5% BSA. The PKH26-labeled rASC-sEVs were obtained by centrifuging at 100 000 × *g* for 70 min. The pellets were further purified by re-suspension in PBS and centrifuging at 100 000 × *g* for 70 min to remove the redundant PKH26. The total amount of rASC-sEVs was represented as the fluorescence value of labeled rASC-sEVs detected by the Thermo Scientific Microplate Reader (Thermo, USA). After loading the labeled rASC-sEVs onto PLLA, PDA-PLLA and DSPE-PLLA, respectively, the wound dressings were placed in a 48-well plate containing 200 μl PBS and incubated in 37°C. All 200 μl PBS was sucked out and its fluorescence value was detected as the released amount of rASC-sEVs. The release rate was calculated according to the following equation: release rate (%) = *F_t_*/*F*_0_ × 100% (*F*_0_: initial fluorescence value, *F_t_*: release fluorescence value at time *t*).

### rASC-sEV uptake assay

Fibroblasts, keratinocytes and macrophages were seeded on various fibrous wound dressings, respectively. After 24 h, the cells were fixed with 4% paraformaldehyde (Leagene, China) for 30 min, followed by incubating with the Cell Navigator F-actin Labeling Kit (Abcam, USA) for 1 h and DAPI (Beyotime Biotechnology, China) for 5 min and observed under the confocal laser scanning microscope (CLSM, Leica TCS SP8, Germany).

### Cell migration

CytoSelect™ 24-Well Wound Healing Assay Kit from Cell Biolabs Inc. (San Diego, CA, USA) was used to study the effects of different wound dressings on the migration of fibroblasts *in vitro*. Briefly, sEVs@PDA-PLLA and sEVs@DSPE-PLLA were placed in a 24-well plate, and the cell migration inserts were put upon the wound dressings. A total of 12 × 10^4^ fibroblasts stained with Cell Tracker Green CMFDA (Shanghai Yisheng, China, 1:1000) were seeded on the wound dressings. The inserts were removed after 24 h. The migration of fibroblasts was observed with Axio Observer 7 (Carl Zeiss, Germany) at 0, 24 and 48 h. The migration rate was calculated according to the following equation: migration rate (%) = *S_t_*/*S*_0_ × 100% (*S*_0_: initial gap area, *S*_t_: cell covered area amid the gap at time *t*).

### Cell proliferation

Cell proliferation was determined using Cell Counting Kit-8 (CCK-8; Dojindo Laboratories, Japan) according to the protocol of the manufacturer on Days 1, 3 and 5. In brief, 4 × 10^4^ keratinocytes and 2 × 10^4^ fibroblasts were cultured on various wound dressings in a 48-well plate, respectively. After washing three times with PBS, the cells were incubated in the culture medium containing 10% CCK-8 reagent at 37°C for 2 h. The absorbance was measured at 450 nm.

### Real-time quantitative polymerase chain reaction

The relative gene levels in fibroblasts and macrophages were determined using quantitative real-time quantitative polymerase chain reaction (RT-qPCR). Total RNA was extracted and reverse-transcribed into cDNA using a Prime Script RT reagent kit with gDNA Eraser (TaKaRa Biotechnology, Japan). The RT-qPCR was performed with the Quant Studio 6 Flex system (Life Technologies, USA) using Maxima SYBR Green/ROX qPCR Master Mix (Thermo Scientific, USA). All genes were normalized to GAPDH (*n* = 4). Gene expression was quantified using the ΔΔCt method, and the fold change was calculated using the formula 2^−ΔΔCt^. The primer sequences used in this study are listed in Tables 1 and 2.

**Table 1.** The primer sequences of macrophages used in this study

Gene	RAW264.7	
	Forward (5′–3′)	Reverse (5′–3′)
IL-1β	TCCAGGATGAGGACATGAGCAC	GAACGTCACACACCAGCAGGTTA
TNF-α	ACTCCAGGCGGTGCCTATGT	GTGAGGGTCTGGGCCATAGAA
ARG1	TGACCGCCGTCGTGTTACTTTA	TTCTCGCCCACTAGGCAGTTC
IL-10	GCCAGAGCCACATGCTCCTA	GATAAGGCTTGGCAACCCAAGTAA
CD206	AGAGCTGGCGAGCATCAAGAG	TTCCATAGGTCAGTCCCAACCAA
GAPDH	GGCACAGTCAAGGCTGAGAATG	ATGGTGGTGAAGACGCCAGTA

**Table 2.** The primer sequences of fibroblasts used in this study

Gene	Fibroblast	
	Forward (5′–3′)	Reverse (5′–3′)
Col I	ATGCCGCGACCTCAAGATG	ATGCCGCGACCTCAAGATG
Col III	AGGTCCTGCGGGTAACACT	AGGTCCTGCGGGTAACACT
VEGF	CTCATCAGCCAGGGAGTCTG	AGGAGCAACCTCTCCAAACC
HIF-1α	TCTCATTTAGAGGCCTGGCT	ATGCATTGGTGAAATGCTGGG
α-SMA	ACCCAGATTATGTTTGAGA	CCGTCAGGCAGTTCGTAG
TGF-β1	CC GAGAAGCGGTACCTGAAC	GGTTGCTGAGGTATCGCCAG
GAPDH	CC GGCACAGTCAAGGCTGAGAAG	ATGGTGGTGAAGACGCCAGTA

### Induction of type I diabetic rat model

All animal procedures were approved by the Animal Care and Use Committee of South China University of Technology. Healthy Sprague-Dawley rats weighing 250–300 g were purchased from the experimental animal center of South China University of Technology, Guangzhou. Diabetes was induced by intravenously injecting 1% streptozotocin (STZ, 6.5 mg/100 g) dissolved in 0.1 M sodium citrate buffer. After 7 days, when the rats showed symptoms of polydipsia, polyphagia, polyuria and weight loss and the blood glucose value of the rats was higher than 16.7 M, it indicated that the model of type I diabetes was successfully induced. All rats were kept in a specific pathogen-free environment and were fed a standard diet.

### The treatment of full-thickness skin wounds in diabetic rats with sEVs@DSPE-PLLA

The rats were anesthetized with isoflurane via a respiratory anesthesia machine and operated under sterile conditions. After shaving and sterilization, two circular, full-thickness skin defect wounds (Φ = 18 mm) were created on the back of each rat. The wounds were randomly divided into four groups, which either received treatment with PLLA, DSPE-PLLA and sEVs@DSPE-PLLA wound dressings or were untreated (blank). Finally, 3M transparent plaster (3M Health Care, Germany) was used to cover the wound surface. All rats were fed in individual cages after surgery.

### The measurement of wound-healing rate

The repairing status of the wounds was observed and recorded using a digital camera on Days 0, 3, 7 and 14 after surgery. The wound-healing rate was calculated according to the following formula: wound-healing rate (%) = (*A*_0_ − *A_t_*)/*A*_0_ × 100%, where *A*_0_ represented the initial wound area on Day 0 and *A*_t_ represented the wound area on Day *t*. The wound area was calculated by analyzing the wound photos with ImageJ software.

### Histological and immunohistochemical analysis

The tissue samples were collected and embedded in OCT (Q800, TA, USA). Five-micrometer-thick cross-sections were cut from the OCT-embedded samples, followed by fixation with cold acetone. Hematoxylin and eosin (HE) staining and Masson staining were performed for histological evaluations. The immunohistochemical staining of Ki67 (GB111141, Servicebio, 1:500), ARG1 (bs-23837r, Bioss, 1:500), iNOS (GB1119, Servicebio, 1:500) and CD31 (GB12063, Servicebio, 1:500) was performed to determine the proliferative cells, macrophage polarization and the formation of new vessels, respectively. The expression levels of iNOS and ARG1were further quantified by calculating the mean optical density via ImagePro Plus 6.0 software. Specifically, the integrated optical density (IOD) of the positive area (Area) of each image was measured and accumulated to obtain the IOD SUM. The mean optical density = (IOD SUM)/Area, which reflected the immunoreaction intensity in this image.

### Statistical analysis

Three independent experiments and at least triplicate samples per group were performed to assure repeatability and statistical significance. Statistical analysis was done with GraphPad Prism 6 software (GraphPad). Data are presented as the mean ± standard deviation. One-way analysis of variance followed by Dunnett's multiple comparisons test was used to determine significant differences between experimental groups. A *P*-values of <0.05 were considered statistically significant.

## Results

### Characterization of rASC-sEVs

**Figure 2.**
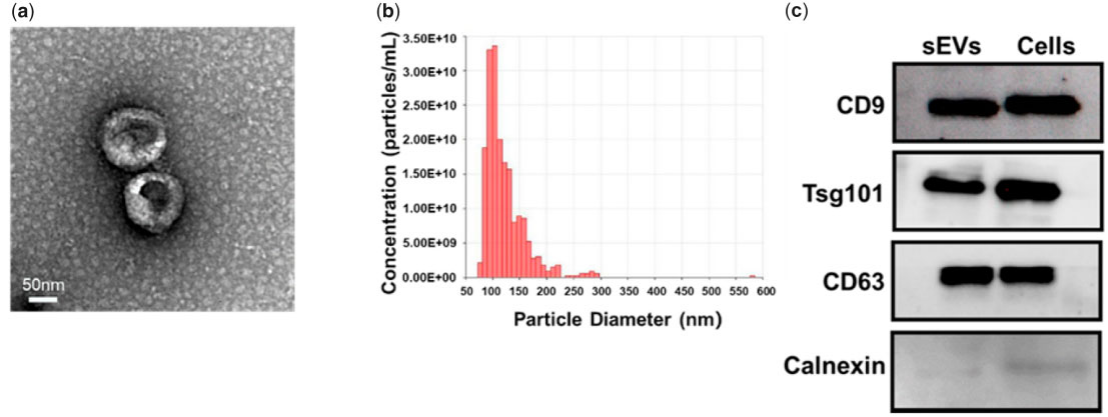
Characterization of the rASC-sEVs. (**a**) Representative TEM images of rASC-sEVs. (**b**) Size distribution of rASC-sEVs. (**c**) Western blot analysis of sEV markers (CD9, CD63, Tsg101) and non-sEV marker (calnexin) in rASC-sEVs and rASCs.

The morphology of the isolated particles from the supernatant of rat adipose-derived mesenchymal stem cells (rASCs) culture showed a typical cup or sphere shape by TEM (Fig. 2a). NTA revealed that the diameter of the nanoparticles was 50–200 nm with a peak at 110 nm (Fig. 2b), indicating the presence of sEVs. The expression of sEV-specific markers CD9, CD63 and Tsg101, together with the negative expression of non-sEV marker Calnexin, in the isolated nanoparticles, confirmed their identity as sEVs (Fig. 2c). All of the above results demonstrated that sEVs from rASCs (rASC-sEVs) were successfully isolated.

### Characterization of various electrospun fibrous wound dressings

**Figure 3.**
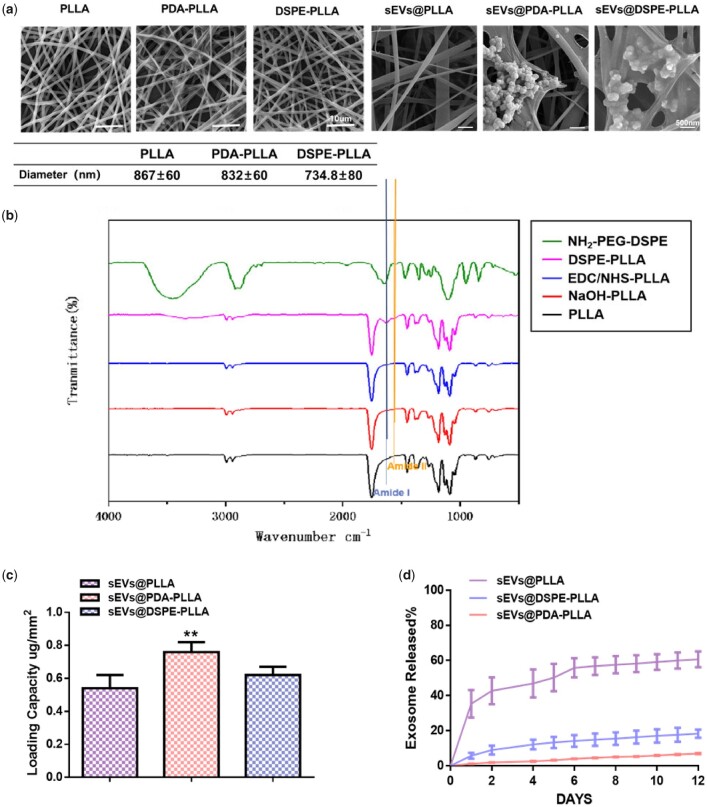
Characterization of various electrospun fibrous wound dressings. (**a**) Representative SEM images and the quantitative analysis of the electrospun PLLA, PDA-PLLA, DSPE-PLLA, sEVs@PLLA, sEVs@ PDA-PLLA and sEVs@ DSPE-PLLA. (**b**) FTIR spectra of DSPE-PLLA. (**c**) The sEVs’ loading capacity of PLLA, PDA-PLLA and DSPE-PLLA. (**d**) The release rate of labeled rASC-sEVs on PLLA, PDA-PLLA and DSPE-PLLA (*statistically significant, **P < 0.01 vs PLLA).

The diameters of PLLA, PDA-PLLA and DSPE-PLLA were 867.7 ± 60, 832.7 ± 60 and 734.8 ± 80 nm, respectively. There was no significant difference in diameter among them, which was consistent with previous reports [37]. The formation of amide was a sign that the amino group of DSPE-PEG_5000_-NH_2_ successfully reacted with the carboxyl group on the surface of PLLA. Specifically, the peak of amide I at about 1650–1660/cm is due to stretching vibrations of C–O groups, and the peak of amide II in the range of 1540–1555/cm originates from stretching C\\N and bending N\\H [38]. The FTIR results showed the peak signals of amide I and amide II were generated at 1650–1660/cm and 1540–1555/cm (Fig. 3b), indicating that DSPE-PEG_5000_-NH_2_ was successfully grafted onto the surface of PLLA.

As shown in Fig. 3a, there was no obvious attachment of sEVs on the fibers in the sEVs@PLLA group due to the low affinity of PLLA fibers for sEVs. Both sEVs@PDA-PLLA and sEVs@DSPE-PLLA groups had clusters of sEVs attached to the fibers, indicating a strong sEVs adhesion of the modified fibers. The loading capacities of rASC-sEVs on PLLA, PDA-PLLA and DSPE-PLLA were 0.54 ± 0.08, 0.76 ± 0.06 and 0.62 ± 0.05 μg/mm^2^, respectively (Fig. 3c). Apparently, PDA-PLLA loaded more rASC-sEVs than PLLA, which might be due to the strong adhesion ability of PDA. There was no significant difference between the quantity of rASC-sEVs loaded on DSPE-PLLA and PLLA.

Figure 3d demonstrates that rASC-sEVs exhibited different kinetic release profiles on PLLA, PDA-PLLA and DSPE-PLLA. A bust release of 35% sEVs was detected during the initial 24 h in the PLLA group. Meanwhile, only 6% sEVs were released from the DSPE-PLLA group. The sEVs loaded on PDA-PLLA were barely released. After 12 days, <40% of sEVs were left on PLLA, while approximately 80% sEVs were retained on DSPE-PLLA. The release rate of sEVs on PDA-PLLA was the slowest and retained almost 90% after 12 days.

### Effects of various electrospun fibrous wound dressings on fibroblasts

**Figure 4.**
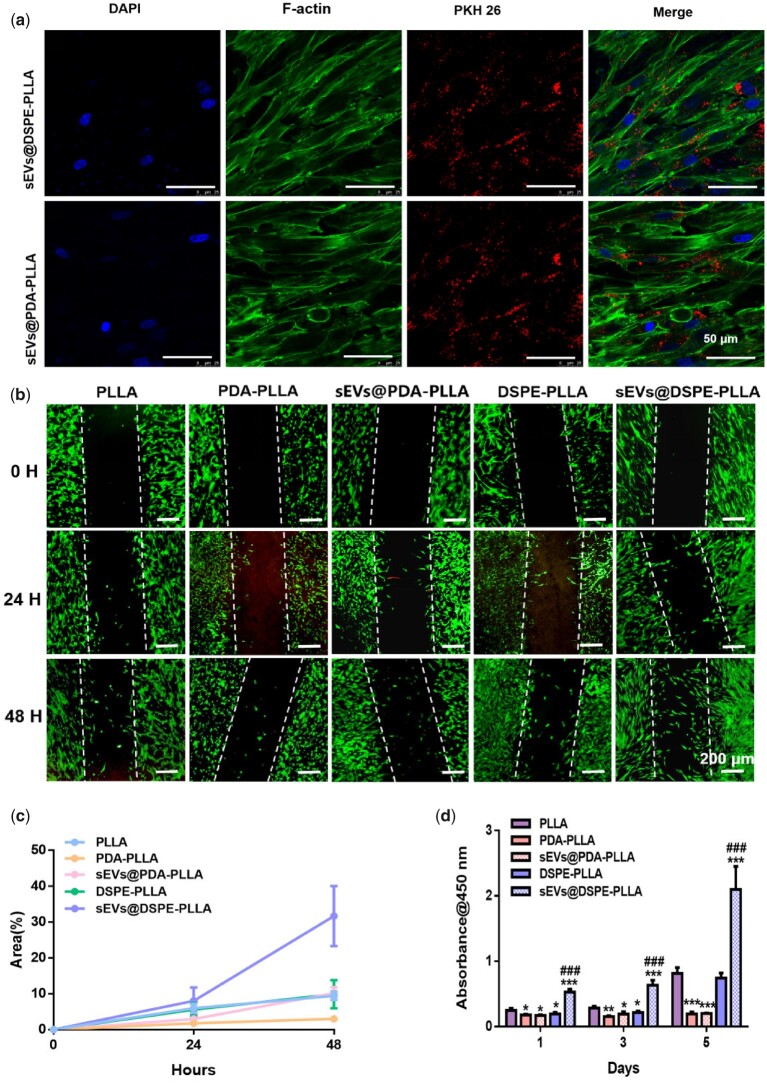
Effects of various electrospun wound dressings on fibroblast migration and proliferation. (**a**) Uptake of PKH26-labeled rASC-sEVs by fibroblasts. (**b**) Representative migration images of fibroblasts cultured for 0, 24 and 48 h. (**c**) Quantitative analysis of the migration rate of fibroblasts. (**d**) Fibroblast proliferation on Days 1, 3 and 5. (*statistically significant, *P < 0.05, **P < 0.01, ***P < 0.001 vs PLLA, ^###^*P* < 0.001 vs DSPE-PLLA).

The uptake of sEVs by fibroblasts was determined by culturing fibroblasts on sEVs@PDA-PLLA and sEVs@DSPE-PLLA for 24 h, respectively. The fluorescence microscopy analysis showed that the majority of rASC-sEVs on both PDA-PLLA and DSPE-PLLA were internalized by fibroblasts, evidenced by the cytoplasmic location of rASC-sEVs (Fig. 4a).

For the migration rate, fibroblasts barely migrated on all scaffolds during the initial 24 h (Fig. 4b). By 48 h, fibroblasts on sEVs@DSPE-PLLA migrated the fastest with a migration rate of 31.7 ± 8.38% (Fig. 4c), followed by sEVs@PDA-PLLA (10.2 ± 1.6%), DSPE-PLLA (9.9 ± 3.92%) and PLLA (9.45 ± 1.19%). Fibroblasts in the PDA-PLLA group showed a suppressed migration rate of 3 ± 0.72% compared to the PLLA group.

Fibroblasts cultured on sEVs@DSPE-PLLA exhibited a higher proliferation rate compared to those on PLLA and DSPE-PLLA (Fig. 4d). Surprisingly, significant inhibition of fibroblast proliferation was observed in the PDA-PLLA and sEVs@PDA-PLLA groups.

**Figure 5.**
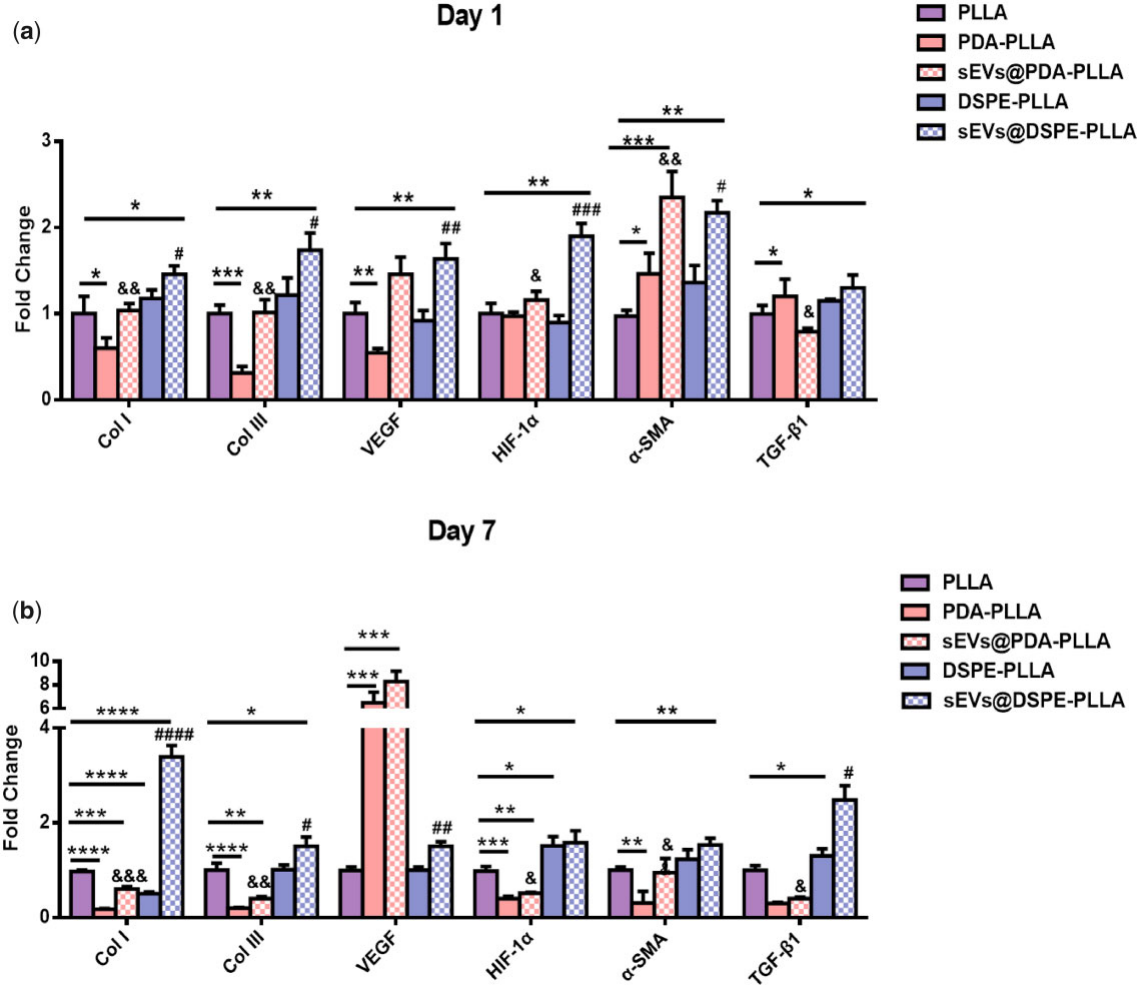
Gene expression of fibroblasts on various electrospun wound dressings on Day 1 (**a**) and Day 7 (**b**) (*statistically significant, *P < 0.05, **P < 0.01, ***P < 0.001, ****P < 0.0001 vs PLLA, ^#^*P* < 0.05, ^##^*P* < 0.01, ^###^*P* < 0.001 vs DSPE-PLLA, ^&^*P* < 0.1, ^&&^*P* < 0.01 vs PDA-PLLA).

Concerning the expression of genes related to wound healing, the levels of Col I, Col III, VEGF, HIF-1α and α-SMA were greatly upregulated in fibroblasts on sEVs@DSPE-PLLA compared to those on PLLA or DSPE-PLLA on Day 1 (Fig. 5a). In addition, the expression of TGF-β1 in fibroblasts on sEVs@DSPE-PLLA tended to be higher. This higher trend continued for the rest of 7 days (Fig. 5b). The expression of genes of Col I, Col III, HIF-1α and α-SMA was greatly upregulated in fibroblasts on sEVs@PDA-PLLA compared to those on PDA-PLLA on Days 1 and 7, while that of Col I, Col III and HIF-1α was significantly inhibited on PDA-PLLA and sEVs@PDA-PLLA on Day 7 compared to PLLA. Surprisingly, the expression of VEGF was significantly higher in fibroblasts on PDA-PLLA and sEVs@PDA-PLLA than in other groups on Day 7.

### Effects of various fibrous wound dressings on keratinocytes

**Figure 6.**
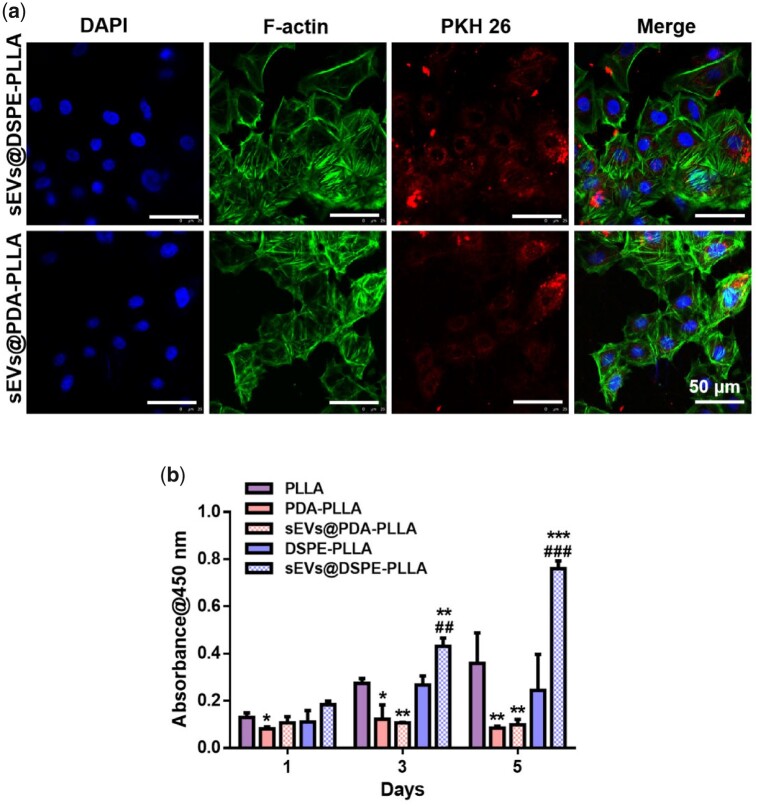
Effects of various electrospun fibrous wound dressings on keratinocytes. (**a**) Uptake of PKH26-labeled rASC-sEVs by keratinocytes. (**b**) Keratinocyte proliferation on Days 1, 3 and 5 (*statistically significant, *P < 0.05, **P < 0.01, ***P < 0.001 vs PLLA, ^##^*P* < 0.01, ^###^*P* < 0.001 vs DSPE-PLLA).

Like fibroblasts, keratinocytes were also capable of uptaking sEVs efficiently. As shown in Fig. 6a, almost all rASC-sEVs were internalized by keratinocytes, distributed in the cytoplasm of keratinocytes.

Compared to PLLA and DSPE-PLLA, sEVs@DSPE-PLLA stimulated keratinocyte proliferation on Days 3 and 5 (Fig. 6b). However, significant inhibition of keratinocyte proliferation was observed on PDA-PLLA and sEVs@PDA-PLLA compared to PLLA, and this trend continued for the rest of 5 days.

### Effects of various electrospun wound dressings on macrophages

**Figure 7.**
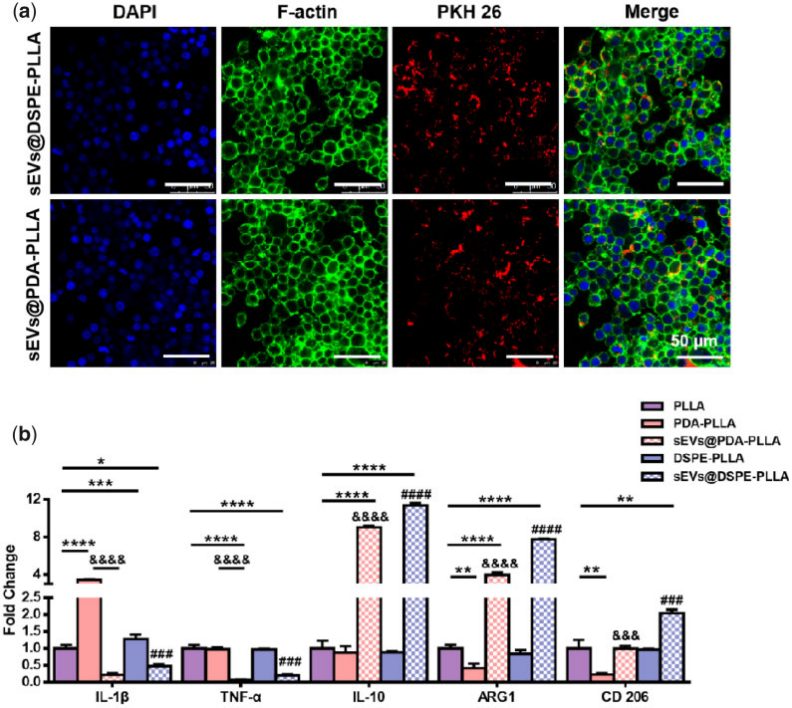
Effects of various electrospun wound dressing on macrophages. (**a**) Uptake of PKH26 labeled rASC-sEVs by macrophages. (**b**) Gene expression of macrophages cultured for 2 days (*statistically significant, *P < 0.05, **P < 0.01, ***P < 0.001, ****P < 0.0001 vs PLLA, ^#^*P* < 0.05, ^##^*P* < 0.01, ^###^*P* < 0.001, ^####^*P* < 0.0001 vs DSPE-PLLA, ^&^*P* < 0.05, ^&&^*P* < 0.01, ^&&&^*P* < 0.001, ^&&&&^*P* < 0.0001 vs PDA-PLLA).

As expected, macrophages uptook rASCs-sEVs immobilized on both PDA-PLLA and DSPE-PLLA (Fig. 7a). Besides, sEVs@PDA-PLLA and sEVs@DSPE-PLLA inhibited the expression of pro-inflammatory cytokines IL-1β and TNF-α, while stimulating the anti-inflammatory cytokines IL-10 and Arginase 1 (ARG1). Besides, the expression of CD 206 in macrophages was upregulated on sEVs@ DSPE-PLLA, which indicated that macrophages on both sEVs@PDA-PLLA and sEVs@DSPE-PLLA exhibited an anti-inflammatory M2 phenotype rather than pro-inflammatory M1 phenotype (Fig. 7b).

### Wound-healing rate in diabetic rats

**Figure 8.**
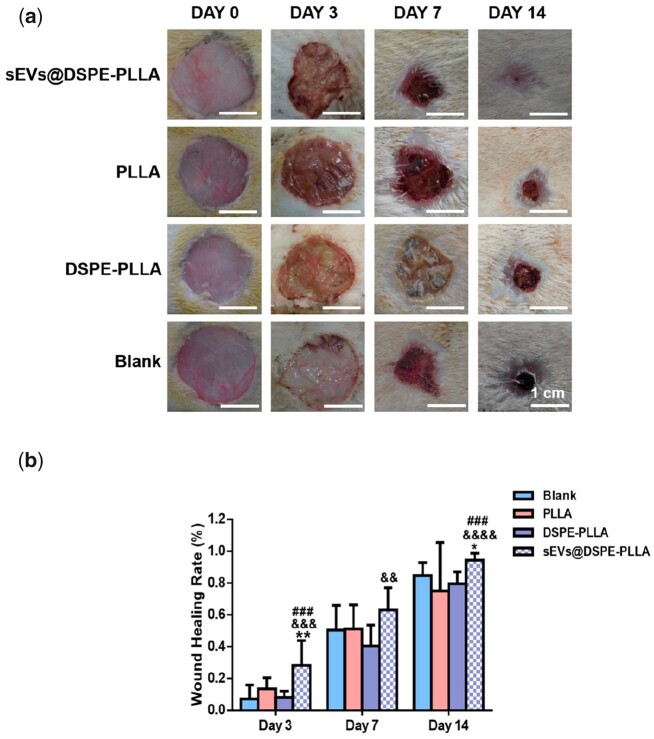
The wound-healing rate in diabetic rats treated with different wound dressings. (**a**) Representative images of the wound beds. (**b**) The wound closure rates on Days 3, 7 and 14 (*n* ≥ 4) (*statistically significant, *P < 0.05, **P < 0.01 vs PLLA, ^&&^*P* < 0.01, ^&&&^*P* < 0.001, ^&&&&^*P* < 0.001 vs DSPE-PLLA, ^###^*P* < 0.001 vs blank).

Because of the inhibitory effects of sEVs@PDA-PLLA on the proliferation of both fibroblasts and keratinocytes, we only explored the effects of sEVs@DSPE-PLLA on wound healing in diabetic rats. Representative images of the full-thickness wounds treated with different wound dressings on Days 0, 3, 7 and 14 were taken (Fig. 8a), which clearly achieved a faster, better healing outcome in wounds treated with sEVs@DSPE-PLLA. Specifically, 3 days after surgery, a significantly shrunk wound was observed in the wound treated with sEVs@DSPE-PLLA. After 7 days, >60% wound closure was achieved in the sEVs@DSPE-PLLA group, followed by the PLLA (51.1%), blank (50.4%) and DSPE-PLLA (40.4%) groups. The wound size in all groups was significantly reduced after 14 days, especially in the sEVs@DSPE-PLLA group, with a 94% wound closure achieved (Fig. 8b).

### Histological analysis of the wounds in diabetic rats

**Figure 9.**
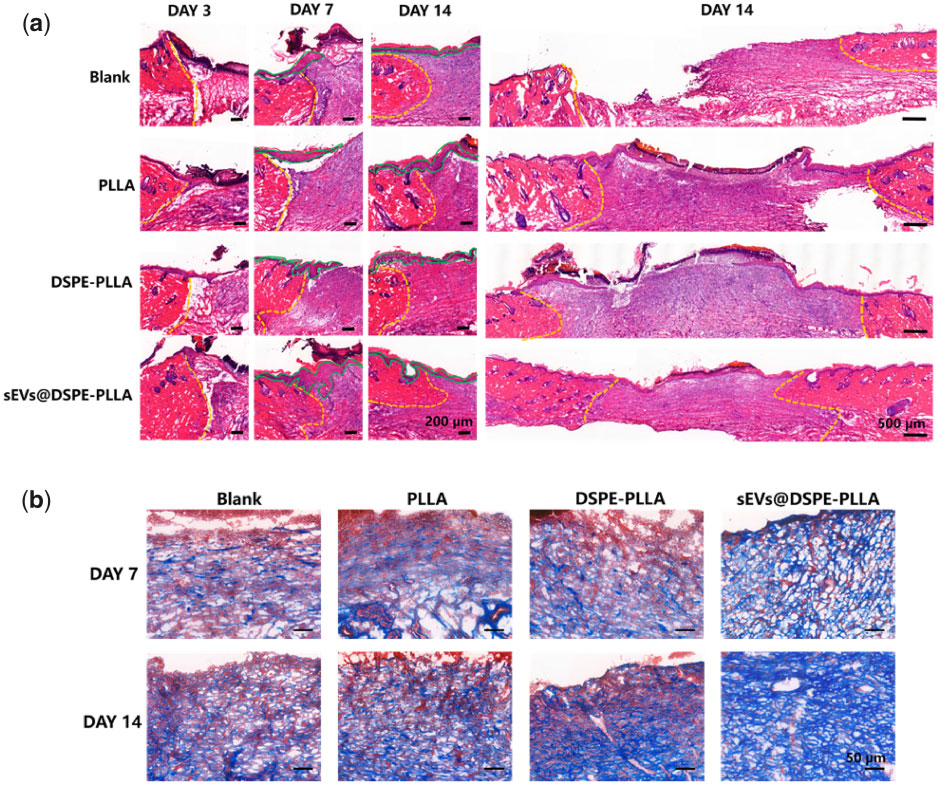
Histological analysis of the wounds in diabetic rats. (**a**) HE staining images of wound tissues on Days 3, 7 and 14. The yellow dotted lines are the boundary between the wound and normal tissue, and the green solid lines outline the epidermal tongues. (**b**) Representative images of Masson’s trichrome staining on Days 7 and 14.

The results of HE staining are shown in Fig. 9a. On Day 3, the wound beds in all groups were filled with fibrin clots with a large number of inflammatory cells in all groups except the blank. This might be due to the fact that the electrospun micro/nanofibers provided a temporary matrix to assist cells to migrate. On Day 7, typical epidermal tongues (green line) could be seen in each group, indicating keratinocytes at wound edges were migrating to the center. On Day 14, the newly regenerated epidermis almost fully covered the wound site with a neat structure in the sEVs@DSPE-PLLA group. However, the re-epithelization was severely delayed in other groups, especially in the blank group. According to the results of Masson’s trichrome staining (Fig. 9b), collagen fibers were detected in all groups on Day 7, with most collagen being deposited in the sEVs@DSPE-PLLA group. On Day 14, the deposited collagen fibers in each group increased, especially in the sEVs@DSPE-PLLA group. Moreover, collagen fibers in the sEVs@DSPE-PLLA group were arranged more orderly. Our results showed that sEVs@DSPE-PLLA accelerated the re-epithelization and collagen deposition during the wound-healing process.

### Immunohistochemical analysis of the wounds in diabetic rats

**Figure 10.**
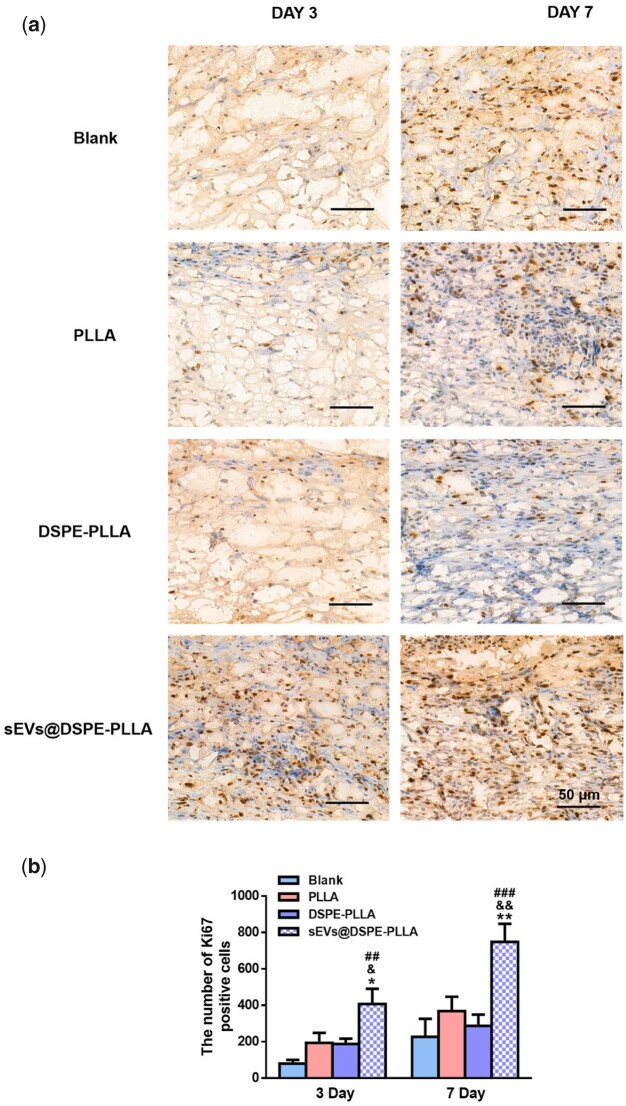
Cell proliferation in the wounds of diabetic rats. (a) Immunohistochemical staining of Ki67 expression at Days 3 and 7. (**b**) Quantification of the Ki67-positive cells (*statistically significant, *P < 0.05, **P < 0.01 vs PLLA, ^&^*P* < 0.05, ^&&^*P* < 0.01 vs DSPE-PLLA, ^##^*P* < 0.01 vs blank).

Ki67 immunohistochemical staining was performed on Days 3 and 7 to detect the proliferating cells in the wound beds. As shown in Fig. 10a, Ki67-positive cells were observed in the wound beds of all groups on Day 3, with the sEVs@DSPE-PLLA group being the most. Consistently, the quantitative analysis showed that the sEVs@DSPE-PLLA group had 408 ± 83 cells, followed by the PLLA group and the DSPE-PLLA group, which had 194 ± 55 cells and 188 ± 29 cells, respectively. The number of proliferating cells in the blank group is the least, and only about 80 ± 20 cells were detected (Fig. 10b). On Day 3, some Ki67-positive cells are not fibroblastic-like cells, and they may be some immune cells, such as neutrophils and macrophages. But on Day 7, while some Ki67-positive immune cells were still detected in the blank, PLLA and DSPE-PLLA groups, the majority of Ki67-positive cells in the sEVs@DSPE-PLLA were fibroblasts. On Day 7, the number of Ki67-positive cells in the sEVs@DSPE-PLLA group notably increased, much higher than that in all other groups.

The immunohistochemical staining of ARG1 and iNOS was performed on Days 3 and 7 to determine the macrophage phenotypes (Fig. 11). The expression levels of ARG1 were higher on Day 7 than on Day 3 in all groups. Apparently, higher ARG1 expression was detected in the sEVs@DSPE-PLLA group compared to the other three groups on both Days 3 and 7 (Fig. 11a and c). Conversely, the lowest expression of iNOS was detected in sEVs@DSPE-PLLA on Days 3 and 7 (Fig. 11b and c). On Day 7, the expression of iNOS in sEVs@DSPE-PLLA was further reduced to a nearly undetectable level. However, the expression of iNOS in the blank, PLLA and DSPE groups was still high, indicating that the wound beds were still in an inflammatory environment.

**Figure 11.**
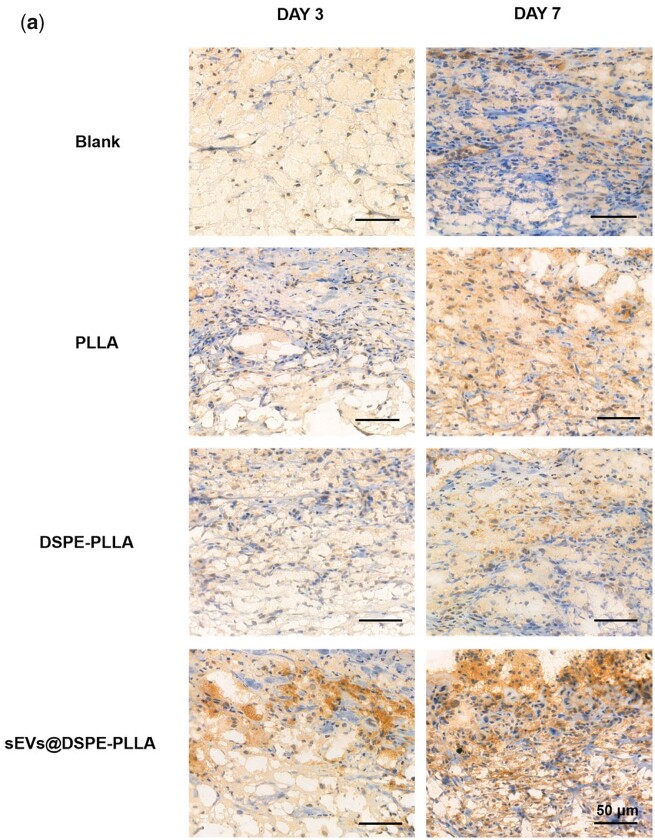
Immunohistochemical staining of ARG1 (**a**) and iNOS (**b**) in macrophages in the wounds of diabetic rats on Days 3 and 7. (**c**) Quantification of ARG1 and iNOS in macrophages in the wounds of diabetic rats on Days 3 and 7.

**Figure 12.**
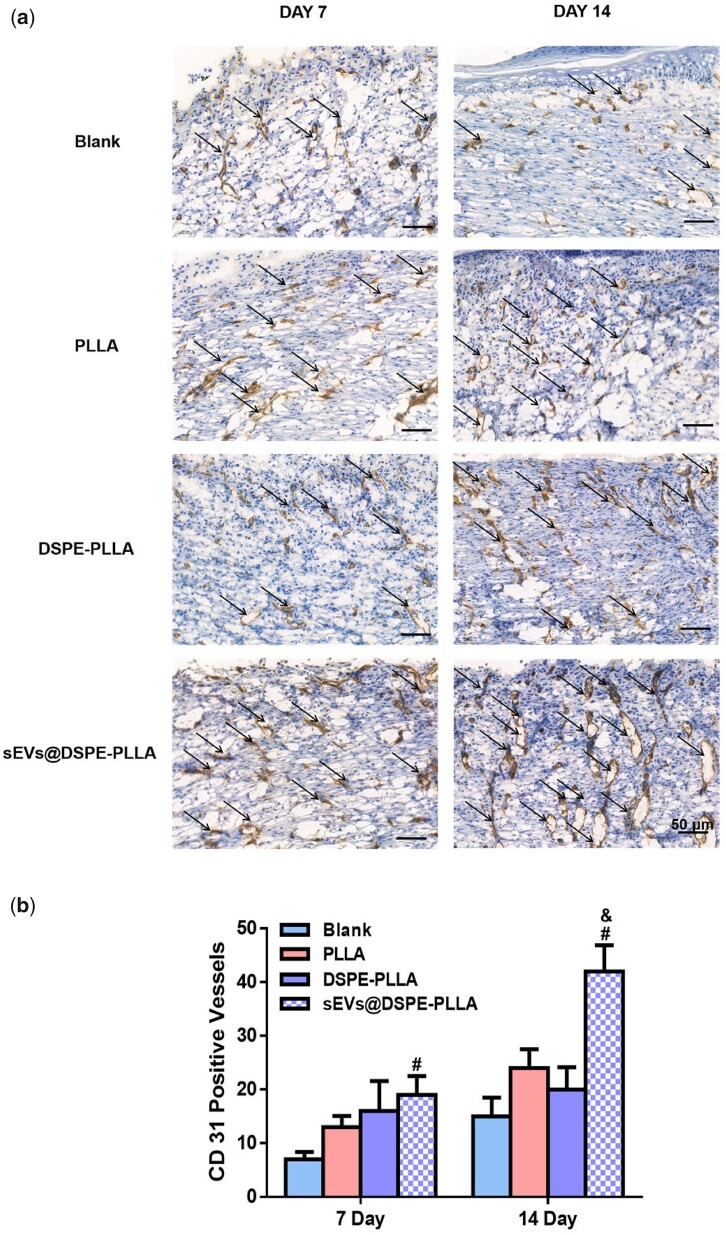
Immunohistochemical staining of CD31 expression (**a**) and the quantitative analysis (**b**) in wounds of diabetic rats on Days 7 and 14. Endothelial cells are indicated by arrows (*statistically significant, **P < 0.05 vs PLLA, ^&&^*P* < 0.01 vs DSPE-PLLA, ^#^*P* < 0.05, ^##^*P* < 0.01 vs blank).

In addition, the expression of CD31 on Days 7 and 14 was examined to illustrate the angiogenesis at the wound sites (Fig. 12). The number of newly formed microvessels in the sEVs@DSPE-PLLA group was the largest (19 ± 3.53) among all groups, followed by DSPE-PLLA (16 ± 5.65) and PLLA (13 ± 2.12). The microvessels in the blank group were the least (7 ± 1.41). On Day 14, significantly more microvessels formed in all groups. Similar to the results on Day 7, the largest number of microvessels was observed in the sEVs@DSPE-PLLA group (41.5 ± 4.94).

**Figure 11.**
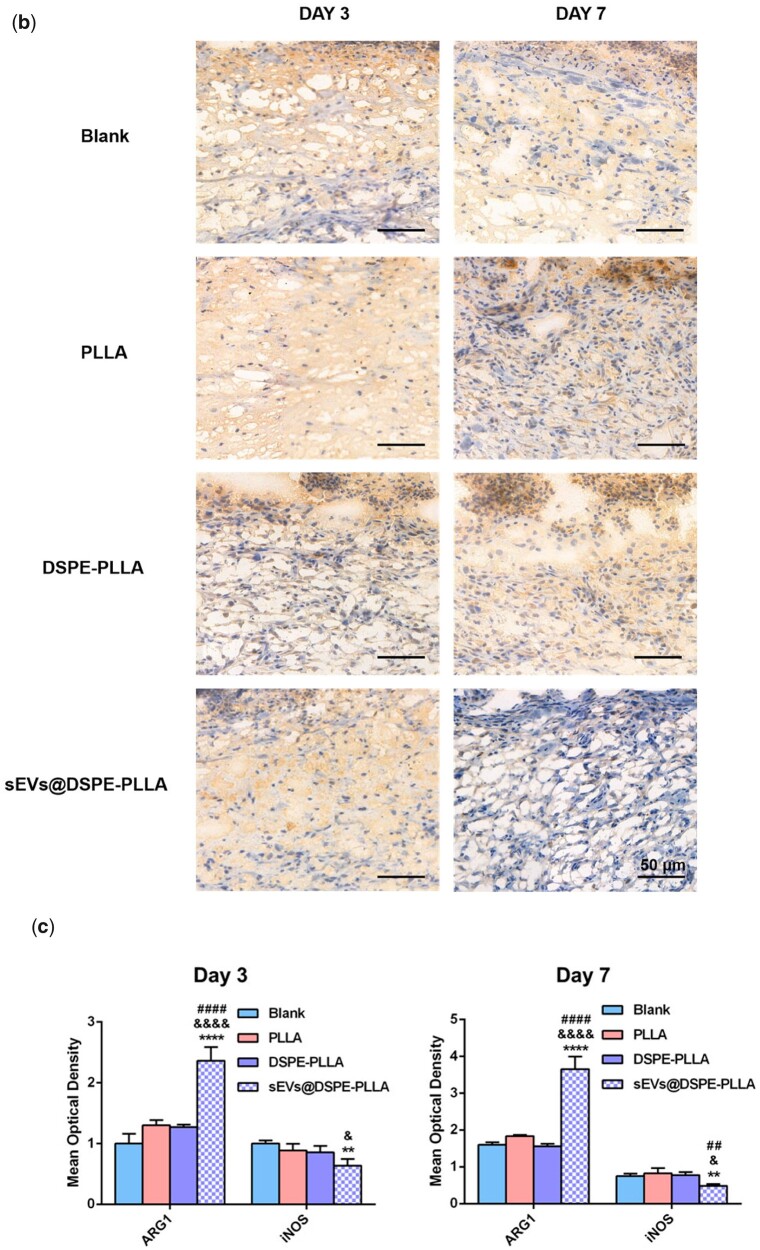
(Continued).

## Discussion

To exert optimal therapeutical efficacy of sEVs, a local and slow-release pattern that mimics the paracrine function of parent cells should be achieved. Several strategies have been developed to improve the local retention and release profile of sEVs. For example, chemical conjugation realizes the long-term binding of sEVs to the substrates. However, it may impair the functions of sEVs due to the alteration in the modified surface proteins. Therefore, mild tethering methodologies that can anchor sEVs on the wound dressings without compromising their integrity and functionality are in high demand [39]. Li et al. [40] constructed a laminin-derived peptide PPFLMLLKGSTR-modified HA hydrogel (pGel), which can capture exosomes through the integrins on the membrane surface of exosomes. Li et al. [40] designed a novel bio-specific peptide (TKKTLPTAHLHNRS), which specifically bind to type I collagen scaffolds with its N-terminal heptapeptide (TKKTLRT) and the transferrin receptor of exosomes with the other segment (AHLHNRS). It is noted that these strategies used nature receptors of exosomes as anchor points. Such occupation of the nature receptors may impair the functions of extracellular vesicles, considering that many nature receptors mediate intracellular communication across a wide range of physiological and pathophysiological settings. PDA, which is highly adhesive for biological reagents, is supposed to be able to anchor extracellular vesicles as well. For example, Li et al. [41] coated PLGA scaffolds with PDA to enable a slow local release of exosomes. Similarly, we also found that PDA modification effectively improved the sEVs’ loading capacity of PLLA and greatly slowed the release rate of sEVs from PLLA. Besides, sEVs@PDA-PLLA stimulated the VEGF expression in fibroblasts (Fig. 5) and helped the M2 polarization of macrophages (Fig. 7b). However, PDA modification exhibited remarked inhibitory effects on cell proliferation and migration (Figs 4 and 6). Previous studies have shown that PDA-modified surfaces inhibited the proliferation and migration of vascular smooth muscle cells probably through the negative regulation of catechol on cell growth and motility [42], but further investigation is required to elucidate how PDA affects cell behaviors. It is also worth mentioning that many conflict results regarding the effects of PDA on cell proliferation exist, indicating the interaction between PDA and cell is cell type dependent. Another strategy we developed in this study was to modify the electrospun PLLA fibers with phospholipids, which can easily insert into the lipid bilayer of sEV membrane as a result of the hydrophobic interaction between lipids, thereby achieving the retention and sustained release of sEVs on the PLLA fibers. As expected, DSPE-PLLA showed improved sEVs’ loading and retention capacities compared to PLLA (Fig. 3c and d). Unlike chemical conjugation, the anchoring of phospholipids into the sEV membrane is non-covalent and reversible; thus, the anchored phospholipids dissociate from the membrane constantly, which explains the faster release rate of sEVs from DSPE-PLLA than that of from PDA-PLLA. One critical factor that determines the dissociation rate is the degree of the interaction force between the phospholipids and the membrane, which depends on the properties of the phospholipids. For example, phospholipids with double oleyl chains, such as DSPE used in this study, exhibit a stronger hydrophobic interaction than those with single oleyl chain [43]. Thus, it is possible to adjust the sEVs’ release profile by using different phospholipids. Besides, since no surface proteins are required for the insertion of DSPE into the sEV membrane, the biological functions of sEVs are unlikely impaired. Indeed, sEVs@DSPE-PLLA effectively regulates multiple key players involved in skin repair, including fibroblasts, keratinocytes and macrophages, and enhances the wound-healing process in diabetic rats.

Wound healing is a multifaceted process governed by sequential yet overlapping phases, including hemostasis, inflammation, proliferation and remodeling [28]. The inflammatory phase begins almost immediately after injury. Prompt resolution of inflammation is considered beneficial to wound repair. In contrast, a wound trapped in a persistent inflammatory state usually leads to chronic wounds. One of the most important immune cells during the inflammatory phase is macrophages, which can roughly be divided into two groups: the M1 phenotype that stimulates the secretion of pro-inflammatory cytokines and the M2 phenotype that promotes cell division and collagen synthesis in the wound bed [44]. In the microenvironment of chronic wound healing, most macrophages maintain the M1 phenotype, resulting in the excessive secretion of inflammatory factors [45]. According to our results, sEVs@DSPE-PLLA alleviated the inflammatory response by promoting the expression of anti-inflammatory genes (ARG1, CD 206, IL-10) and inhibiting the expression of pro-inflammatory genes (IL-1β, TNF-α) in macrophages *in vitro* (Fig. 6). Consistently, sEVs@DSPE-PLLA drove a switch in the macrophage phenotype from M1 to M2 in the wounds of diabetic rats, as evidenced by the notably increased expression of ARG1 and the decreased expression of iNOS 7 days after surgery (Fig. 10). This phenotypic transformation was mainly regulated by sEVs as DSPE-PLLA did not show similar effects. Studies have shown that sEVs derived from ASCs not only induce the transition of macrophages from a pro-inflammatory M1 phenotype to an anti-inflammatory M2 phenotype but also affect the cellular behavior of T cells [24, 46]. This effect may be attributed to the microRNAs enclosed in the sEVs and their roles in intracellular communication. For example, it has been reported that sEVs secreted by MSCs contain miR-223, which suppresses the classic pro-inflammatory pathways by targeting pknox1 [47]. Besides, our previous study detected various immunomodulatory microRNAs, such as miR-132-3p, miR-125-5p, miR-133a-3p and let-7b-5p in rat rASCs-sEVs [48], which may contribute to the regulatory effects of sEVs on macrophage polarization.

Skin cells at the wound edges play active roles during the proliferation and remodeling phases after the inflammation. For example, fibroblasts migrating into the wound beds and proliferating are critical because they produce a new collagen-rich ECM and secrete a variety of growth factors [49, 50]. Under the stimulation of TGF-β1, fibroblasts differentiate into contractile myofibroblasts, which promote wound closure [51, 52]. Except for fibroblasts, keratinocytes, the most abundant epidermal cells, start to migrate within a few hours after injury to fill the wound defect, which is particularly important because it helps restore the epidermis structure and the barrier function [53]. Our results showed that sEVs@DSPE-PLLA promoted the proliferation of both fibroblasts and keratinocytes *in vitro* (Figs 4 and 6). Consistently, more proliferating fibroblasts and keratinocytes existed in the wound beds of diabetic rats treated with sEVs@DSPE-PLLA 7 days after surgery (Fig. 10). Besides the trophic factors in the sEVs, sEVs may stimulate cell proliferation via their surface proteins. A recent report demonstrates that Wnt3a on the exterior surface of CD63+ exosomes contributes to the promoted fibroblast and endothelial cell proliferation [54]. Our results also showed that sEVs@DSPE-PLLA promoted the remodeling of collagen and wound contraction *in vivo* (Fig. 9). This probably resulted from the increased secretion of collagen and TGF-β1 in fibroblast by sEVs@DSPE-PLLA, together with the enhanced myofibroblastic differentiation of fibroblast, as evidenced by the upregulated gene expression of Col I, Col III, TGF-β and α-SMA (Fig. 5). According to our results (Fig. 9), the newly regenerated epidermis almost fully covered the wound site in the sEVs@DSPE-PLLA group 14 days after the surgery. In contrast, re-epithelialization was not complete in other groups. Similarly, sEVs derived from MSCs have been reported to promote the synthesis and remodeling of ECM and accelerate the re-epithelialization in wound healing [55, 56].

In terms of angiogenesis, sEVs@DSPE-PLLA also stimulated the formation of microvessels in the newly regenerated skin tissue (Fig. 11). Such acceleration in angiogenesis could effectively promote chronic wound healing by providing sufficient nutrients and oxygen to cells. Many proangiogenic microRNAs, including miR-31, miR-125a, miR-130a, miR-210 and miR-126a, have been detected in the sEVs derived from ASCs [48, 57, 58], providing a possible explanation for the promoted angiogenesis by rASC-sEVs.

## Conclusions

In this study, we successfully fabricated phospholipid-grafted PLLA electrospun micro/nanofibers immobilized with sEVs (sEVs@DSPE-PLLA) for wound repair. By slowly releasing sEVs, sEVs@DSPE-PLLA effectively regulated the functions of key cells involved in wound healing, including fibroblasts, keratinocytes and macrophages. Further *in vivo* study revealed that sEVs@DSPE-PLLA enhanced the wound-healing process by resolving inflammation, promoting cell growth and collagen deposition and enhancing angiogenesis at the wound sites in diabetic rats. Our study suggested the potential application of sEVs@DSPE-PLLA as a novel wound dressing to comprehensively improve the unbalanced microenvironment and promoted the repair process of chronic wounds.

## Funding

This work was financially supported by the National Key R&D Program of China (2021YFB3800900), National Natural Science Foundation of China (31971266), the Key Research and Development Program of Guangzhou (202007020002) and Guangdong Province Basic and Applied Research Foundation (2022A1515011925).

*Conflicts of interest statement*. There are no conflicts to declare.

## References

[1] Singer AJ , Clark RA. Mechanisms of disease: cutaneous wound healing. N Engl J Med 1999;341:738–46. 10471461 10.1056/NEJM199909023411006

[2] Nwomeh BC , Yager DR, Cohen IK. Physiology of the chronic wound. Clin Plast Surg 1998;25:341–56. 9696897

[3] Robson MC. Growth factors as wound healing agents. Curr Opin Biotechnol 1991;2:863–7. 1367960 10.1016/s0958-1669(05)80122-3

[4] Wang M , Wang CG, Chen M, Xi YW, Cheng W, Mao C, Xu TZ, Zhang XX, Lin C, Gao WY, Guo Y, Lei B. Efficient angiogenesis-based diabetic wound healing/skin reconstruction through bioactive antibacterial adhesive ultraviolet shielding nanodressing with exosome release. ACS Nano 2019;13:10279–93. 31483606 10.1021/acsnano.9b03656

[5] Wang CG , Wang M, Xu TZ, Zhang XX, Lin C, Gao WY, Xu HZ, Lei B, Mao C. Engineering bioactive self-healing antibacterial exosomes hydrogel for promoting chronic diabetic wound healing and complete skin regeneration. Theranostics 2019;9:65–76. 30662554 10.7150/thno.29766 PMC6332800

[6] Dinh T , Tecilazich F, Kafanas A, Doupis J, Gnardellis C, Leal E, Tellechea A, Pradhan L, Lyons TE, Giurini JM, Veves A. Mechanisms involved in the development and healing of diabetic foot ulceration. Diabetes 2012;61:2937–47. 22688339 10.2337/db12-0227 PMC3478547

[7] Naghibi M , Smith RP, Baltch AL, Gates SA, Wu DH, Hammer MC, Michelsen PB. The effect of diabetes mellitus on chemotactic and bactericidal activity of human polymorphonuclear leukocytes. Diabetes Res Clin Pract 1987;4:27–35. 3121272 10.1016/s0168-8227(87)80030-x

[8] Martin P , Nunan R. Cellular and molecular mechanisms of repair in acute and chronic wound healing. Br J Dermatol 2015;173:370–8. 26175283 10.1111/bjd.13954 PMC4671308

[9] Bauer SM , Bauer RJ, Velazquez OC. Angiogenesis, vasculogenesis, and induction of healing in chronic wounds. Vasc Endovascular Surg 2005;39:293–306. 16079938 10.1177/153857440503900401

[10] Usui ML , Mansbridge JN, Carter WG, Fujita M, Olerud JE. Keratinocyte migration, proliferation, and differentiation in chronic ulcers from patients with diabetes and normal wounds. J Histochem Cytochem 2008;56:687–96. 18413645 10.1369/jhc.2008.951194 PMC2430161

[11] Hu SC , Lan CE. High-glucose environment disturbs the physiologic functions of keratinocytes: focusing on diabetic wound healing. J Dermatol Sci 2016;84:121–7. 27461757 10.1016/j.jdermsci.2016.07.008

[12] Terashi H , Izumi K, Deveci M, Rhodes LM, Marcelo CL. High glucose inhibits human epidermal keratinocyte proliferation for cellular studies on diabetes mellitus. Int Wound J 2005;2:298–304. 16618316 10.1111/j.1742-4801.2005.00148.x PMC7951445

[13] Brem H , Stojadinovic O, Diegelmann RF, Entero H, Lee B, Pastar I, Golinko M, Rosenberg H, Tomic-Canic M. Molecular markers in patients with chronic wounds to guide surgical debridement. Mol Med 2007;13:30–9. 17515955 10.2119/2006-00054.Brem PMC1869625

[14] Chamberlain G , Fox J, Ashton B, Middleton J. Concise review: mesenchymal stem cells: their phenotype, differentiation capacity, immunological features, and potential for homing. Stem Cells 2007;25:2739–49. 17656645 10.1634/stemcells.2007-0197

[15] Murphy MB , Moncivais K, Caplan AI. Mesenchymal stem cells: environmentally responsive therapeutics for regenerative medicine. Exp Mol Med 2013;45:e54. 24232253 10.1038/emm.2013.94 PMC3849579

[16] Wang Y , Han ZB, Song YP, Han ZC. Safety of mesenchymal stem cells for clinical application. Stem Cells Int 2012;2012:1–4. 10.1155/2012/652034 PMC3363282 22685475

[17] Song M , Heo J, Chun J-Y, Bae HS, Kang JW, Kang H, Cho YM, Kim SW, Shin D-M, Choo M-S. The paracrine effects of mesenchymal stem cells stimulate the regeneration capacity of endogenous stem cells in the repair of a bladder-outlet-obstruction-induced overactive bladder. Stem Cells Dev 2014;23:654–63. 24192209 10.1089/scd.2013.0277

[18] Liang X , Ding Y, Zhang Y, Tse H-F, Lian Q. Paracrine mechanisms of mesenchymal stem cell-based therapy: current status and perspectives. Cell Transplant 2014;23:1045–59. 23676629 10.3727/096368913X667709

[19] Phinney DG , Pittenger MF. Concise review: MSC-derived exosomes for cell-free therapy. Stem Cells 2017;35:851–8. 28294454 10.1002/stem.2575

[20] Pitt JM , Kroemer G, Zitvogel L. Extracellular vesicles: masters of intercellular communication and potential clinical interventions. J Clin Invest 2016;126:1139–43. 27035805 10.1172/JCI87316 PMC4811136

[21] Colombo M , Raposo G, Théry C. Biogenesis, secretion, and intercellular interactions of exosomes and other extracellular vesicles. Annu Rev Cell Dev Biol 2014;30:255–89. 25288114 10.1146/annurev-cellbio-101512-122326

[22] Brennan MÁ , Layrolle P, Mooney DJ. Biomaterials functionalized with MSC secreted extracellular vesicles and soluble factors for tissue regeneration. Adv Funct Mater 2020;30:1909125. 32952493 10.1002/adfm.201909125 PMC7494127

[23] Robbins PD , Morelli AE. Regulation of immune responses by extracellular vesicles. Nat Rev Immunol 2014;14:195–208. 24566916 10.1038/nri3622 PMC4350779

[24] Blazquez R , Sanchez-Margallo FM, de la Rosa O, Dalemans W, Alvarez V, Tarazona R, Casado JG. Immunomodulatory potential of human adipose mesenchymal stem cells derived exosomes on in vitro stimulated T cells. Front Immunol 2014;5:556. 25414703 10.3389/fimmu.2014.00556 PMC4220146

[25] Todorova D , Simoncini S, Dignat-George F, Lacroix R, Sabatier F. Extracellular vesicles in angiogenesis. Circ Res 2017;120:1658–73. 28495996 10.1161/CIRCRESAHA.117.309681 PMC5426696

[26] Zhang B , Wang M, Gong A, Zhang X, Wu X, Zhu Y, Shi H, Wu L, Zhu W, Qian H, Xu W. HucMSC‐exosome mediated‐Wnt4 signaling is required for cutaneous wound healing. Stem Cells 2015;33:2158–68. 24964196 10.1002/stem.1771

[27] Henriques-Antunes H , Cardoso RMS, Zonari A, Correia J, Leal EC, Jimenez-Balsa A, Lino MM, Barradas A, Kostic I, Gomes C, Karp JM, Carvalho E, Ferreira L. The kinetics of small extracellular vesicle delivery impacts skin tissue regeneration. ACS Nano 2019;13:8694–707. 31390518 10.1021/acsnano.9b00376

[28] Wang PH , Huang BS, Horng HC, Yeh CC, Chen YJ. Wound healing. J Chin Med Assoc 2018;81:94–101. 29169897 10.1016/j.jcma.2017.11.002

[29] Barnes CP , Sell SA, Boland ED, Simpson DG, Bowlin GL. Nanofiber technology: designing the next generation of tissue engineering scaffolds. Adv Drug Deliv Rev 2007;59:1413–33. 17916396 10.1016/j.addr.2007.04.022

[30] Venugopal J , Low S, Choon AT, Ramakrishna S. Interaction of cells and nanofiber scaffolds in tissue engineering. J Biomed Mater Res B: Appl Biomater 2008;84:34–48. 17477388 10.1002/jbm.b.30841

[31] Zuk PA , Zhu M, Mizuno H, Huang J, Futrell JW, Katz AJ, Benhaim P, Lorenz HP, Hedrick MH. Multilineage cells from human adipose tissue: implications for cell-based therapies. Tissue Eng 2001;7:211–28. 11304456 10.1089/107632701300062859

[32] Lee H , Dellatore SM, Miller WM, Messersmith PB. Mussel-inspired surface chemistry for multifunctional coatings. Science 2007;318:426–30. 17947576 10.1126/science.1147241 PMC2601629

[33] Teramura Y , Iwata H. Cell surface modification with polymers for biomedical studies. Soft Matter 2010;6:1081–91.

[34] Teramura Y , Iwata H. Bioartificial pancreas: microencapsulation and conformal coating of islet of langerhans. Adv Drug Deliv Rev 2010;62:827–40. 20138097 10.1016/j.addr.2010.01.005

[35] Sakurai K , Teramura Y, Iwata H. Cells immobilized on patterns printed in DNA by an inkjet printer. Biomaterials 2011;32:3596–602. 21353304 10.1016/j.biomaterials.2011.01.066

[36] Théry C , Amigorena S, Raposo G, Clayton A. Isolation and characterization of exosomes from cell culture supernatants and biological fluids. Curr Protoc Cell Biol 2006;30:3:22–9. 3 10.1002/0471143030.cb0322s30 18228490

[37] Guo Z , Genlong J, Huang Z, Li H, Ge Y, Wu Z, Yu P, Li Z. Synergetic effect of growth factor and topography on fibroblast proliferation. Biomed Phys Eng Express 2020;6:065036. 10.1088/2057-1976/abc8e2 34035190

[38] Sadeghi AR , Nokhasteh S, Molavi AM, Khorsand-Ghayeni M, Naderi-Meshkin H, Mahdizadeh A. Surface modification of electrospun PLGA scaffold with collagen for bioengineered skin substitutes. Mater Sci Eng C: Mater Biol Appl 2016;66:130–7. 27207046 10.1016/j.msec.2016.04.073

[39] Zhang L , Fan C, Hao W, Zhuang Y, Liu X, Zhao Y, Chen B, Xiao Z, Chen Y, Dai J. NSCs migration promoted and drug delivered exosomes‐collagen scaffold via a bio‐specific peptide for one‐step spinal cord injury repair. *Adv Healthcare Mater* **2021**;10:2001896. Doi: 10.1002/adhm.202001896. 33522126

[40] Li L , Zhang Y, Mu J, Chen J, Zhang C, Cao H, Gao J. Transplantation of human mesenchymal stem-cell-derived exosomes immobilized in an adhesive hydrogel for effective treatment of spinal cord injury. Nano Lett 2020;20:4298–305. 32379461 10.1021/acs.nanolett.0c00929

[41] Li W , Liu Y, Zhang P, Tang Y, Zhou M, Jiang W, Zhang X, Wu G, Zhou Y. Tissue-Engineered bone immobilized with human adipose stem cells-derived exosomes promotes bone regeneration. ACS Appl Mater Interfaces 2018;10:5240–54. 29359912 10.1021/acsami.7b17620

[42] Luo R , Tang L, Zhong S, Yang Z, Wang J, Weng Y, Tu Q, Jiang C, Huang N. In vitro investigation of enhanced hemocompatibility and endothelial cell proliferation associated with quinone-rich polydopamine coating. ACS Appl Mater Interfaces 2013;5:1704–14. 23384031 10.1021/am3027635

[43] Kato K , Itoh C, Yasukouchi T, Nagamune T. Rapid protein anchoring into the membranes of mammalian cells using oleyl chain and poly(ethylene glycol) derivatives. Biotechnol Prog 2004;20:897–904. 15176897 10.1021/bp0342093

[44] Mantovani A , Sozzani S, Allavena P, Locati M, Sica A. Macrophage polarization: tumor-associated macrophages as a paradigm for polarized M2 mononuclear phagocytes. Trends Immunol 2002;23:549–55. 12401408 10.1016/s1471-4906(02)02302-5

[45] Diegelmann RF , Evans MC. Wound healing: an overview of acute, fibrotic and delayed healing. Front Biosci 2004;9:283–9. 14766366 10.2741/1184

[46] Lo Sicco C , Reverberi D, Balbi C, Ulivi V, Principi E, Pascucci L, Becherini P, Bosco MC, Varesio L, Franzin C, Pozzobon M, Cancedda R, Tasso R. Mesenchymal stem cell-derived extracellular vesicles as mediators of anti-inflammatory effects: endorsement of macrophage polarization. Stem Cells Transl Med 2017;6:1018–28. 28186708 10.1002/sctm.16-0363 PMC5442783

[47] He X , Dong Z, Cao Y, Wang H, Liu S, Liao L, Jin Y, Yuan L, Li B. MSC-derived exosome promotes M2 polarization and enhances cutaneous wound healing. Stem Cells Int 2019;2019:7132708. 31582986 10.1155/2019/7132708 PMC6754952

[48] Ji Y , Han W, Fu X, Li J, Wu Q, Wang Y. Improved small extracellular vesicle secretion of rat adipose-derived stem cells by microgrooved substrates through upregulation of the ESCRT-III-associated protein alix. Adv Healthcare Mater 2021;10:2100492. 10.1002/adhm.202100492 34176241

[49] Martinez-Ferrer M , Afshar-Sherif AR, Uwamariya C, Crombrugghe BD, Davidson JM, Bhowmick NA. Dermal transforming growth factor-beta responsiveness mediates wound contraction and epithelial closure. Am J Pathol 2010;176:98–107. 19959810 10.2353/ajpath.2010.090283 PMC2797873

[50] Kim WJ , Mohan RR, Mohan RR, Wilson SE. Effect of PDGF, IL-1alpha, and BMP2/4 on corneal fibroblast chemotaxis: expression of the platelet-derived growth factor system in the cornea. Invest Ophthalmol Vis Sci 1999;40:1364–72. 10359318

[51] Leask A , Abraham DJ. TGF-β signaling and the fibrotic response. FASEB J 2004;18:816–27. 15117886 10.1096/fj.03-1273rev

[52] Jeon YK , Jang YH, Yoo DR, Kim SN, Lee SK, Nam MJ. Mesenchymal stem cells' interaction with skin: wound-healing effect on fibroblast cells and skin tissue. Wound Repair Regen 2010;18:655–61. 20955344 10.1111/j.1524-475X.2010.00636.x

[53] Falanga V. Wound healing and its impairment in the diabetic foot. Lancet 2005;366:1736–43. 16291068 10.1016/S0140-6736(05)67700-8

[54] McBride JD , Rodriguez-Menocal L, Guzman W, Candanedo A, Garcia-Contreras M, Badiavas EV. Bone marrow mesenchymal stem cell-derived CD63+ exosomes transport Wnt3a exteriorly and enhance dermal fibroblast proliferation, migration, and angiogenesis in vitro. Stem Cells Dev 2017;26:1384–98. 28679315 10.1089/scd.2017.0087

[55] Kim Y-J , Yoo S, Park HH, Lim HJ, Kim Y-L, Lee S, Seo K-W, Kang K-S. Exosomes derived from human umbilical cord blood mesenchymal stem cells stimulates rejuvenation of human skin. Biochem Biophys Res Commun 2017;493:1102–8. 28919421 10.1016/j.bbrc.2017.09.056

[56] Liu K , Chen C, Zhang H, Chen Y, Zhou S. Adipose stem cell-derived exosomes in combination with hyaluronic acid accelerate wound healing through enhancing re-epithelialization and vascularization. Br J Dermatol 2019;181:854–6. 30953591 10.1111/bjd.17984

[57] Liang X , Zhang L, Wang S, Han Q, Zhao RC. Exosomes secreted by mesenchymal stem cells promote endothelial cell angiogenesis by transferring miR-125a. J Cell Sci 2016;129:2182–9. 27252357 10.1242/jcs.170373

[58] Cao J , Xiao R. Exosomes are comparable to source adipose stem cells in fat graft retention with up-regulating early inflammation and angiogenesis. Plast Reconstr Surg 2020;146:503e–4e. 10.1097/PRS.0000000000007201 32639430

